# Biological sex and multilingualism: The interaction of risk and reserve for dementia

**DOI:** 10.1002/alz.087692

**Published:** 2025-01-03

**Authors:** Noelia Calvo, Rumsha Siddiqui, Natalie Phillips, Gillian Einstein

**Affiliations:** ^1^ University of Toronto, Toronto, ON Canada; ^2^ Queen’s University, Kingston, ON Canada; ^3^ Concordia University, Montreal, QC Canada; ^4^ Linkoping University, Linkoping Sweden; ^5^ Rotman Research Institute, Toronto, ON Canada

## Abstract

**Background:**

Globally, females are at twice the risk of AD than males; in Canada, over 700,000 are living with Alzheimer’s disease and related dementia (ADRDs), with 72% being female. However, females maintain verbal memory in the face of more AD pathology than men. It is unclear how multilingualism, considered a resilience factor, might interact with the risk and resilience of sex. Thus, we wondered if female sex and multilingualism might interact to confer more resilience in individuals with Mild Cognitive Impairment (MCI) in a Canadian cohort: The Comprehensive Assessment of Neurodegeneration and Dementia (COMPASS‐ND).

**Method:**

Neuropsychology data from 270 female and male participants diagnosed with MCI were analyzed using female or male sex as a categorical variable with 2 levels: 116 women and 156 men. They self‐reported language use and history allowing a multilingualism score which identified 148 multilinguals, and 124 monolinguals. First univariate analysis was used to explore individual differences among the 4 groups: women, men, monolinguals, multilinguals. Then, different models using Path analysis and Structural Equation Modelling (SEM) were used to create a Cognitive Reserve index (CRI) which accounted for multilingualism and biological sex. Simple regressions were used to estimate cognitive performance in relation to the CRI.

**Result:**

Females with MCI had higher scores than men in The Rey Auditory Verbal Learning Test (RAVLT); this effect was even stronger in those women who had ever used Hormone replacement therapy. Multilinguals outperformed monolinguals in the verbal fluency component of the Delis‐Kaplan Executive Function System (D‐KEFS) which was performed in English. Moreover, CRI significantly predicted increased performance in tasks measuring visuospatial memory and attention.

**Conclusion:**

Previous mixed‐sex studies have suggested that multilingualism may be a proxy for cognitive reserve delaying the onset of AD symptoms for approximately 4‐5 years. Other studies have indicated that women are more affected by AD than men but that verbal memory may be a form of reserve for them. Here, we show that multilingualism and biological sex together may associate with increased neuropsychological performance even in the presence of MCI.

